# Impact of Temperature and Interface Traps on Threshold Voltage and Carrier Mobility of Hydrogen-Terminated Diamond MOSFETs

**DOI:** 10.3390/ma19132791

**Published:** 2026-07-01

**Authors:** Nuwayyir Alshammari, Mulpuri V. Rao, Qiliang Li

**Affiliations:** 1 Department of Electrical Engineering, George Mason University, Fairfax, VA 22030, USA; nalsham5@gmu.edu (N.A.);; 2 Department of Advanced Manufacturing and Robotics, Peking University, Beijing 100871, China

**Keywords:** hydrogen-terminated diamond, MOSFET, interface traps, temperature dependence, threshold voltage, mobility, TCAD simulation

## Abstract

We present a Sentaurus TCAD model calibrated for the hydrogen-terminated diamond p-channel MOSFET reported by Hirama et al. and validated using the published transfer characteristics. We used the calibrated model to analyze threshold voltage (V_th_) and effective mobility (μ_eff_) over a temperature range from 300 to 600 K and an interface-trap density range from 10^12^ to 10^16^ cm^−2^·eV^−1^. The analysis shows three main trends: (i) a drain-bias-dependent shift in V_th_ that becomes stronger at higher temperatures; (ii) a threshold shift toward more negative values with increasing temperature and interface-trap density, showing a coupled thermal trap effect; and (iii) a mobility response that stays near the phonon-limited regime at low trap densities but degrades rapidly as trap density increases. We extracted compact empirical expressions for ΔV_th_ (T, D_it_) and μ_eff_ (T, D_it_) that reproduce the simulated trends with good accuracy for device-level comparison. The results show that interface-trap control is important to achieve thermally stable, high-performance diamond MOSFETs.

## 1. Introduction

Ultra-wide-bandgap (UWBG) semiconductors are promising for high-power and high-temperature electronics. Their large bandgaps and high electric fields enable higher breakdown voltages and faster switching speeds, extending performance beyond conventional technologies [1,2,3]. Among UWBG semiconductors, diamond stands out due to its bandgap of 5.47 eV, thermal conductivity near 2000 W m^−1^ K^−1^, and high carrier mobility. These properties make diamond suitable for power and radio frequency (RF) devices operating in high-temperature harsh environments [4,5,6].

Hydrogen-terminated diamond MOSFETs (HD-MOSFETs) operate through surface-transfer doping at the hydrogen-terminated diamond surface, where a two-dimensional hole gas (2DHG) is formed. This surface channel supports high sheet carrier density and large drain current [7,8,9]. The carrier transport in HD-MOSFETs is limited to only a few nanometers near the diamond interface [10,11]. As a result, the device electrostatics depend on both gate bias and device geometry, as well as on surface charge transfer, interface-trap occupancy, and the vertical electric-field distribution [12,13]. Since the channel is not formed by bulk inversion [14], short-channel effects such as drain-induced barrier lowering (DIBL) should be interpreted with care in HD-MOSFETs.

Recent experimental work has improved the stability of HD-MOSFET channels. NO_2_-induced hole accumulation, stabilized by atomic-layer-deposited (ALD) Al_2_O_3_ passivation, has enabled high-current operation with improved stability [14,15]. TCAD simulations have also been used to examine how gate electrostatics and interface quality affect current drive and transconductance in HD-MOSFETs [16,17]. Gate-dielectric and interface engineering further shows that interface traps and fixed charges can affect threshold voltage and gate leakage. Related studies on novel nanoelectronic and energy-conversion devices also show that interface engineering, defect control, and charge stability strongly influence device behavior [18,19]. These studies show why physics-based modeling is still needed when optimizing diamond MOSFET structures [20,21,22,23,24].

Even with this progress, the temperature-dependent behavior of HD-MOSFETs with interface traps is not yet fully understood. Temperature can change trap occupancy, modify the effective channel charge, shift the threshold voltage (V_th_), and reduce transconductance (g_m_) [25,26,27]. Increased phonon scattering and trap-assisted charge modulation can also reduce the effective field-effect mobility. As a result, device optimization and parameter extraction become more difficult when several mechanisms vary at the same time [28,29,30]. Since these effects are difficult to isolate experimentally, simulation provides a controlled method to vary trap density and temperature independently.

In this work, we calibrated a Sentaurus TCAD model against the HD-MOSFET reported by Hirama et al. [31] and validated it using published transfer characteristics. We used this model to independently vary temperature (300–600 K) and interface-trap density (D_it_ = 10^12^–10^16^ cm^−2^·eV^−1^). This allowed us to isolate trap-induced threshold shifts and mobility degradation from phonon and contact effects. The results quantify how D_it_ and temperature jointly affect the transfer-doped channel without the confounding factors present in experiment [32,33,34,35,36,37].

## 2. Methodology

We performed two-dimensional (2D) device simulations using Synopsys Sentaurus TCAD (Synopsys, Inc., Sunnyvale, CA, USA) to examine the hydrogen-terminated diamond p-channel MOSFET (HD-MOSFET). The device geometry and bias conditions replicate the experimental structure reported by Hirama et al. [31] and the calibrated model of Wong et al. [38].

**Figure 1:**
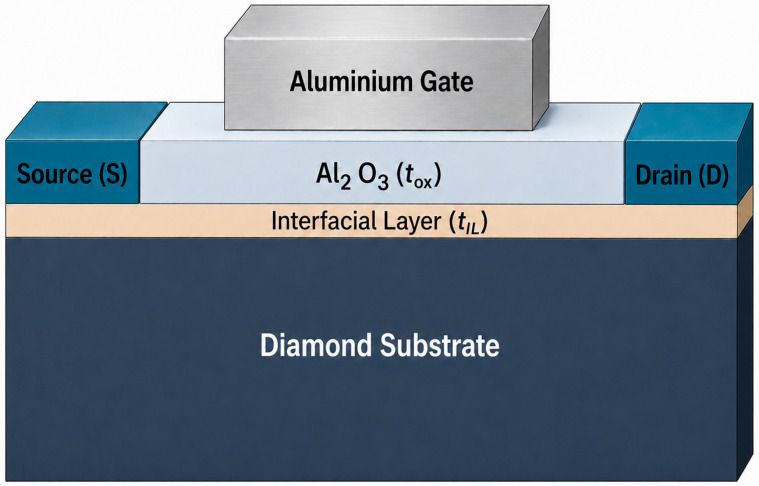
Cross-sectional schematic of the simulated HD-MOSFET structure, showing the aluminum gate, source and drain contacts, Al_2_O_3_ gate dielectric (t_ox_ = 17 nm), interfacial layer (t_IL_ = 0.2 nm), and diamond substrate.

The device structure (Figure 1) has a 17 nm Al_2_O_3_ gate dielectric on a hydrogen-terminated diamond channel. The gate length is L_G_ = 0.4 μm, with lateral source-gate and gate–drain spacings of approximately L_GS_ ≈ L_GD_ ≈ 1 μm. An explicit interfacial layer (IL) is included between the Al_2_O_3_ gate dielectric and the hydrogen-terminated diamond channel, with a thickness of t_IL_ = 0.2 nm. Interface effects are added using fixed interfacial charges at the Al_2_O_3_/IL and IL/diamond interfaces under the gate, following the calibrated “device 1” simulation approach of Wong et al. [38].

Source and drain contacts are modeled as ohmic gold (Au) contacts, with a terminal contact resistance of R_c_ = 2.2 Ω⋅mm per contact, consistent with reported experimental values. The diamond buffer layer has unintentional boron doping with an activation energy of 0.36 eV, to reflect realistic background impurity levels. Thermal boundary conditions are implemented by fixing the bottom surface of the structure at 300 K to represent ideal heat sinking. This boundary condition serves only as a thermal reference and does not restrict the simulated lattice temperature during temperature-dependent analyses.

### 2.1. Physics and Extraction

This section summarizes the physical models used in the simulations and the procedures applied for electrical parameter extraction. Carrier transport is described using a temperature-dependent mobility model that includes phonon scattering, Coulomb scattering, and normal-field mobility degradation to capture surface-confined transport in the hydrogen-terminated channel. Recombination and charge trapping are modeled using Shockley–Read–Hall (SRH) statistics.

The threshold voltage (V_th_) is mainly extracted from transfer characteristics using the Y-function as the primary method. To improve numerical robustness near the transconductance peak, the transconductance is smoothed and the Y-function is examined within a sliding gate overdrive window. For consistency with prior HD-MOSFET studies, the extracted V_th_ values are cross-validated using a constant-current criterion of ∣I_D_∣ = 1 mA/mm. Gate-voltage sweeps are performed at fixed drain biases of V_D_ = −12, −10, −6, −3, −2, and −1 V to examine drain-bias-induced electrostatic effects and the resulting dependence of V_th_ on V_D_.

### 2.2. Calibration

**Figure 2:**
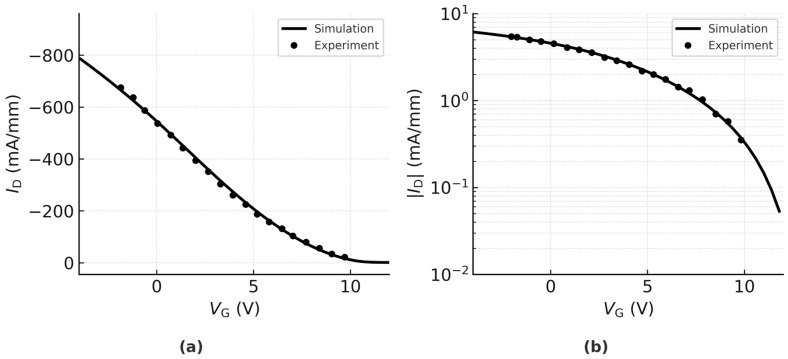
Calibrated transfer characteristics at V_D_ = −10 V: (**a**) linear-scale I_D_–V_G_ showing experiment (markers) and simulation (solid line); (**b**) semi-log ∣I_D_∣–V_G_ for the same dataset, indicating agreement in the subthreshold regime and near turn-off.

The simulation is validated against the experimental transfer characteristics reported by Hirama et al. [31]. At V_D_ = −10 V, the simulated transfer curve closely reproduces the experimental data on both linear and semilogarithmic scales, as shown in Figure 2a,b. The extracted threshold voltages differ by only 0.606 V (V_th_^exp^ ≈ 8.306 V, V_th_^sim^ ≈ 8.912 V), using the constant-current threshold voltage at ∣I_D_∣ = 1 mA/mm.

On the semilogarithmic scale, the current fit gives RMSE_log_ ≈ 0.014 decades and MAE_log_ ≈ 0.010 decades (approximately 3% and 2.3%, respectively). The simulated minimum leakage current is reduced to 0.0534 mA/mm, compared with 0.290 mA/mm in experiment at ∣V_GS_∣ ≈ 10.2 V.

This good agreement in threshold voltage and current magnitude confirms that the calibrated model captures the essential electrostatic and transport characteristics of the reported device. This validation gives a reliable foundation for subsequent analysis of interface-trap and temperature-dependent effects.

### 2.3. Impact of Interface Traps Under Drain Bias

**Figure 3:**
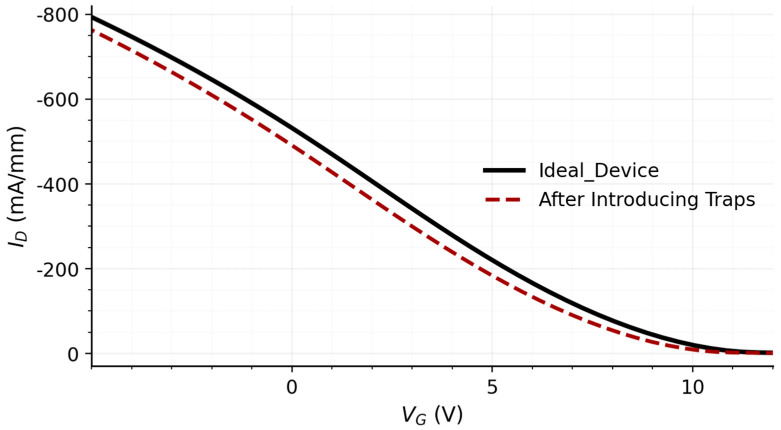
Trap sensitivity at V_D_ = −10 V: ideal, trap-free device (solid) and a trap-included device with D_it_ = 1 × 10^13^ cm^−2^·eV^−1^ (dashed). The presence of interface traps shifts V_th_ to higher V_GS_, reduces on-current, and degrades the subthreshold slope, consistent with interface-charge screening and enhanced Coulomb scattering.

We compared the trap-free device against a representative case that includes interface traps at V_D_ = −10 V, as shown in Figure 3. The interface traps are modeled as acceptor-like states located at the Al_2_O_3_/diamond interface, where the transfer-doped two-dimensional hole gas (2DHG) forms.

When occupied, these states become negatively charged. The energy distribution is assumed to follow a Gaussian profile, extending from the valence-band edge into the bandgap. States located near the valence-band edge have the strongest influence on the surface potential due to their higher occupation probability under operating bias conditions. For the trap-related case shown in Figure 3, a representative interface-trap density of D_it_ = 1 × 10^13^ cm^−2^·eV^−1^ is selected, which lies within the range investigated in this study.

The presence of negatively charged interface states partially screens the applied gate electric field and reduces the effective hole density in the surface channel. As a result, the transfer characteristics exhibit (i) a positive shift in the extracted threshold voltage, (ii) a reduction in drain current across the applied gate-voltage range, and (iii) degradation of the subthreshold swing. These trends reflect weakened electrostatic gate control and enhanced Coulomb scattering associated with interface charges.

These results show the important role of interface traps in modulating surface electrostatics and transport in HD-MOSFETs. Accurate modeling of these effects is therefore needed for analyzing switching behavior and assessing device robustness, particularly under elevated-temperature operation where trap occupancy becomes strongly temperature-dependent.

### 2.4. Trap Models and Ranges

To evaluate device stability and trap-induced performance degradation, we introduced interface traps with density D_it_ (cm^−2^·eV^−1^) at the Al_2_O_3_/diamond interface. The trap states are modeled using a Gaussian energy distribution extending from the valence-band edge into the bandgap, consistent with surface-transfer-doped channel configuration.

For the threshold-voltage analysis, selected D_it_ values were 1 × 10^12^, 2 × 10^12^, 5 × 10^12^, and 1 × 10^13^ cm^−2^·eV^−1^. For the mobility analysis, we extended the D_it_ range up to 1 × 10^16^ cm^−2^·eV^−1^, since mobility degradation is likely to become clearer under more severe interface conditions.

The selected D_it_ range was chosen to connect improved H-diamond interfaces with trap-rich sensitivity conditions. Low interface state density has been observed in recent H-terminated diamond FETs where gate stack structure has been optimized, such as D_it_ = 3.19 × 10^11^ cm^−2^·eV^−1^ [39]. On the other hand, low frequency noise analysis of H-terminated diamond FETs showed wide variation in density of traps depending on the trap center and extraction method, with effective GR trap densities varied from (10^12^ cm^−2^) up to (10^18^–10^19^ cm^−2^) [40]. Thus, this range of D_it_ values is relevant for HD-MOSFETs as 2DHG is formed near the oxide/diamond interface, making V_th_ and mobility highly sensitive to interfacial charge.

We performed the temperature-dependent simulations at 300, 400, 500, and 600 K, where both thermal activation and trap occupancy can influence the device behavior. This allows us to quantitatively analyze the trap-induced threshold-voltage shifts and effective mobility degradation under different interface and temperature conditions.

The scope of the TCAD model should also be noted. Trap states were introduced through the use of an idealized energy distribution, allowing us to isolate the effects of the traps individually (D_it_). For practical HD-MOSFETs, there could be more complex energy distributions as well as spatial distributions for the traps, according to the material used and the history of stresses applied to the device. Also, the effect of self-heating during high-power operations was not considered explicitly; rather, only simulations at different temperatures ranging from 300 to 600 K were compared.

## 3. Results and Discussion

### 3.1. Threshold-Voltage Dependence on Drain Bias and Temperature

**Figure 4:**
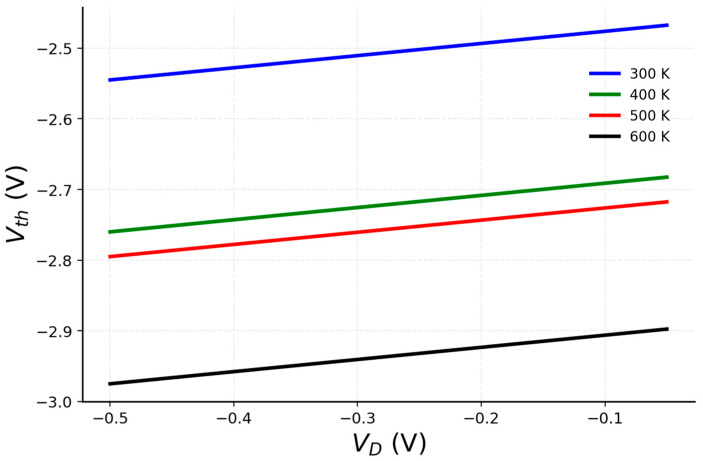
Extracted threshold voltage V_th_ as a function of drain voltage V_D_ for the hydrogen-terminated diamond p-channel MOSFET at temperatures from 300 K to 600 K. Low-magnitude drain bias (∣V_D_∣ ≤ 0.5 V) is used to examine drain–channel electrostatic coupling during threshold extraction under near-linear conditions.

Figure 4 shows the extracted threshold voltage, V_th_, as a function of drain bias, V_D_, for the HD-MOSFETs over a temperature range of 300 to 600 K. Across all drain biases, V_th_ remains negative. This is expected for a p-channel device, where the channel conduction is mainly governed by surface-transfer doping and the formation of a two-dimensional hole gas at the hydrogen-terminated diamond/oxide interface.

As the drain voltage magnitude is reduced, the extracted V_th_ shifts toward less negative values at each temperature. This shift is mostly related to drain-bias-induced electrostatic coupling in the near-threshold regime. Since V_th_ is taken near the onset of channel conduction, small changes in surface potential or interface charge can shift the extracted value. The gate does not cause this shift alone; the drain bias also affects it. In an HD-MOSFET, this changes the interfacial charge balance, which is then reflected in the apparent threshold voltage.

A temperature dependence is also present. As the temperature increases from 300 K to 600 K, V_th_ moves toward more negative values. Interface-charge occupancy and carrier statistics likely account for part of this shift. Transport effects may contribute as well. Temperature also changes trap occupancy, which affects gate control of the channel hole density. At higher temperature, the surface charge near the interface may change, and this change appears in the transfer curves as a shift in the extracted V_th_.

### 3.2. Temperature–Trap Coupling of Δv_th_

**Figure 5:**
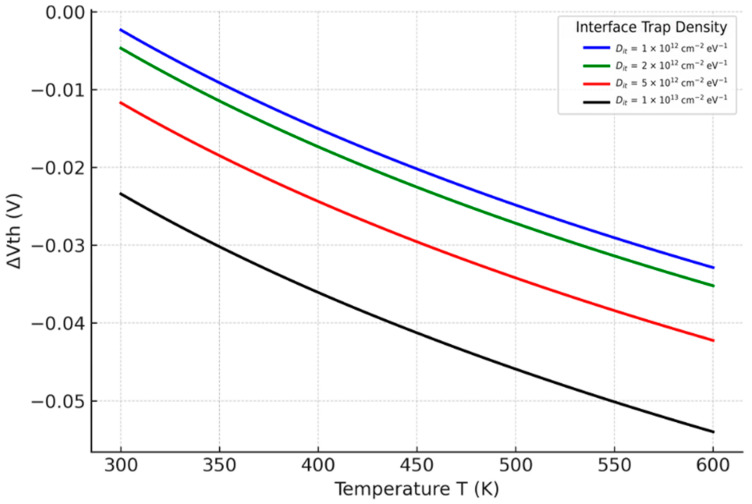
Temperature dependence of the threshold-voltage shift ΔV_th_ at fixed interface-trap densities D_it_. ΔV_th_ becomes more negative with increasing temperature and shows clear curvature.

**Figure 6:**
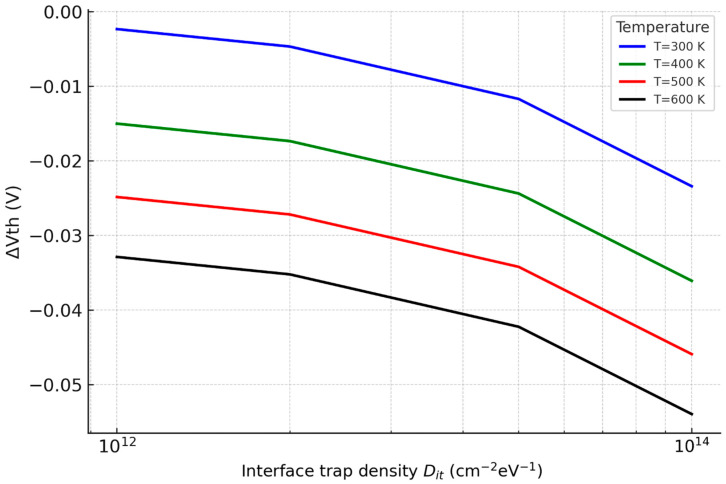
Interface-trap dependence of ΔV_th_ at fixed temperatures. Increasing D_it_ produces an additional negative shift in V_th_, with a stronger slope emerging near the 10^12^–10^14^ cm^−2^·eV^−1^ range.

Figure 5 summarizes ΔV_th_(T) at fixed interface-trap density. With increasing temperature, ∣ΔV_th_∣ becomes larger, but the change is not linear. The curvature points to more than a simple temperature shift. Trap occupation and local charge redistribution are likely part of this behavior. At a fixed temperature, higher D_it_ gives an additional negative shift in V_th_, as shown in Figure 6. This shift becomes more noticeable once D_it_ rises above approximately 10^13^ cm^−2^·eV^−1^.

The behavior in Figure 5 and Figure 6 is difficult to explain using temperature or D_it_ alone. As temperature rises, trap occupancy changes, and the interfacial charge balance shifts with it. With higher D_it_, more trap states can take part in the charge exchange. The shift is better treated as a coupled response to temperature and D_it_.

We used a compact semi-empirical relation to represent the combined dependence:

$$\Delta V_{\mathrm{th}}\;(T,\;D_{\mathrm{it}})\;\approx \;\alpha_{1}\;\ln\;(\frac{T}{T_{0}})\;+\;\alpha_{2}\;D_{\mathrm{it}}\;+\;\alpha_{3}\;{D_{\mathrm{it}}}^{2}\;{\left(\frac{T}{T_{0}}\right)}^{n}$$

where T is the operating temperature, T_0_ = 300 K is the reference temperature, and D_it_ is the interface-trap density. The coefficients α_1_, α_2_, α_3_, and n are empirical fitting terms. They are used to describe the simulated ΔV_th_ behavior over the studied range, not to represent separate microscopic trap mechanisms.

The fit was obtained using constrained least-squares regression, with α_1_ ∈ [−0.2, 0.2] and α_2_ ≤ 0. For this dataset, α_1_ ≈ −4.4 × 10^−2^ V and α_2_ ≈ −2.3 × 10^−15^.

To quantify the effect of the higher-order term, the simplified version with α_3_ = 0 was compared against the full expression containing α_3_, based on the processed extracted threshold shift dataset. The simplified version had an RMSE value of 0.04661 V and a MAE of 0.03953 V, whereas the full version had RMSE and MAE values of 0.04648 V and 0.03969 V, respectively. It was seen that the RMSE improved by merely 1.3 × 10^−4^ V by considering α_3_, with the MAE showing a slight increase. Therefore, the higher-order term was removed from the final expression (α_3_ = 0), to avoid overfitting. After removing this term, the reduced expression still tracks the simulated ΔV_th_ data well. We used it mainly for interpolation and design comparison, not as a microscopic trap model.

The interaction of temperature with traps can be explained with respect to the charge neutrality equation of the hydrogen-terminated diamond/oxide interface. For the device discussed here, the conducting channel is confined near the surface region. As a result, any variations in the occupancy of the interface states have a direct influence on the electrostatic potential and the two-dimensional hole concentration of the transfer-doped channel region. As temperature increases, the occupation probability of the trap states changes, and the effect of the trapped charge cannot be considered as a fixed electrostatic perturbation. The variation in temperature causes the interface charge, channel charge, and the surface potential to change together. This causes the temperature-related voltage shift, and the extent of voltage shift increases with increasing D_it_. At low D_it_, the temperature effect is governed mostly by carrier statistics and phonon transport limitation. With increased D_it_ values, trap-assisted charge control gains significance, resulting in a more prominent temperature threshold-voltage variation. Figure 5 and Figure 6 show that temperature sensitivity increases with D_it_. As the trap density increases, the thermal shift becomes stronger, leading to larger ΔV_th_ change over the simulated range.

**Figure 7:**
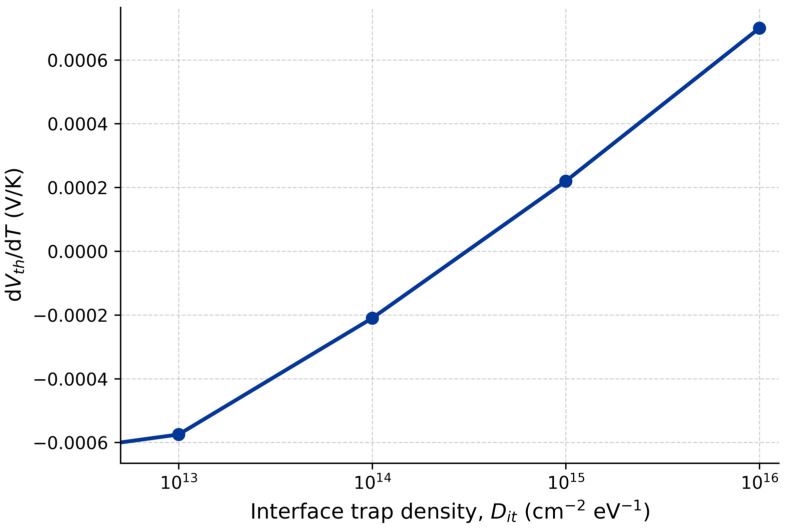
Temperature sensitivity of the threshold voltage dV_th_/dT as a function of interface-trap density D_it_. The clear increase in dV_th_/dT with D_it_ summarizes the coupled temperature–interface-trap effects observed in Figure 5 and Figure 6.

Figure 7 uses the temperature coefficient dV_th_/dT to view the same behavior from another angle. Each point is extracted from an independent linear fit of V_th_ (T) over the simulated temperature range. This gives one temperature-sensitivity value for each D_it_ case.

As D_it_ increases, dV_th_/dT rises monotonically. The threshold voltage becomes more temperature-sensitive when more interface traps are present. The coefficient follows the same temperature–trap trend seen in Figure 5 and Figure 6. For high-temperature HD-MOSFETs, interface quality is not a secondary detail.

### 3.3. Transfer Characteristics at Fixed T and Fixed D_it_

**Figure 8:**
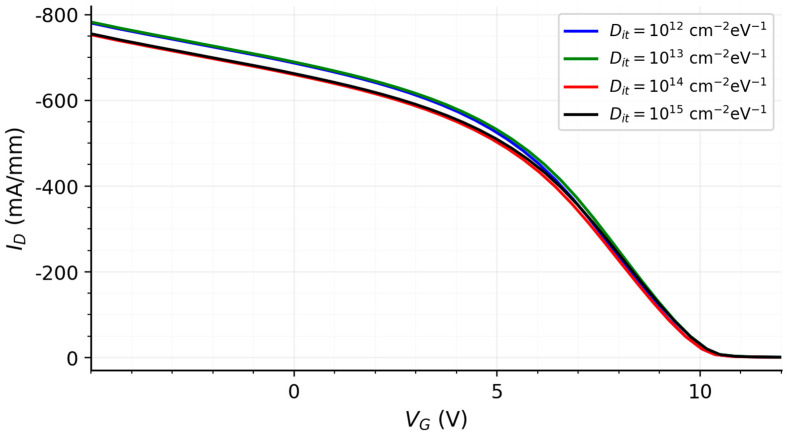
Transfer curves I_D_–V_G_ of the p-channel HD MOSFET at T = 300 K and V_D_ = −6 V for varying interface-trap densities, D_it_, from 10^12^ to 10^15^ cm^−2^·eV^−1^.

Figure 8 compares the I_D_–V_G_ curves at T = 300 K for D_it_ values from 10^12^ to 10^15^ cm^−2^·eV^−1^. On linear scale, the on-state currents stay close across the tested D_it_ range. A small current reduction appears only at the higher trap densities.

The main effect of increasing D_it_ is a shift in the turn-off region toward more positive V_G_. The shift is likely caused by the extra negative charge introduced at the interface by occupied traps. Within this gate-bias window, the drain current at moderate overdrive changes only slightly with D_it_. The threshold region shifts, while the on-state current stays similar across the tested D_it_ range. This suggests that, at room temperature, the electrostatic impact of interface traps is more pronounced near the threshold than in the strong accumulation regime.

**Figure 9:**
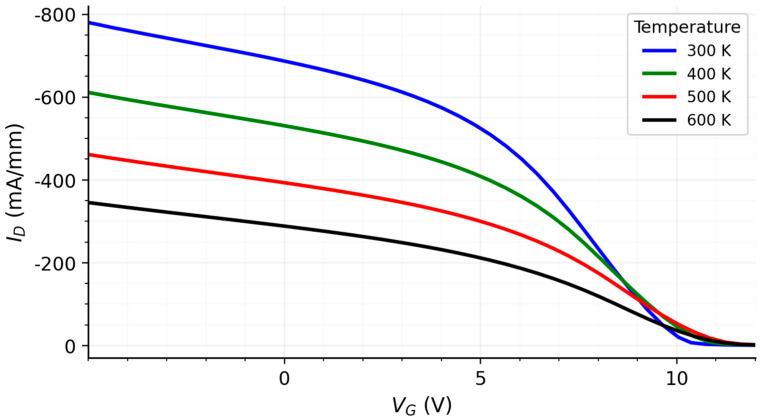
Transfer curves I_D_–V_G_ at D_it_ = 1 × 10^12^ cm^−2^·eV^−1^ with V_D_ = −6 V, for varying temperatures from 300 to 600 K.

In contrast, when D_it_ is fixed at 10^12^ cm^−2^·eV^−1^ and temperature is varied (Figure 9), a clear separation of the transfer characteristics is observed. Increasing temperature leads to a noticeable reduction in on-state current and degradation of the subthreshold slope. This is likely due to enhanced phonon scattering and thermally activated trap occupancy at elevated temperatures.

These observations support using derived electrical metrics, such as transconductance (g_m_) and V_th_. Using the same extraction method helps compare the results across different conditions. The effective field-effect mobility (μ_eff_) is also evaluated at a fixed gate overdrive to separate mobility changes from threshold shifts. These extracted parameters offer enhanced sensitivity to trap- and temperature-induced effects that may not be apparent in the ∣I_D_∣ transfer characteristics.

To further isolate temperature-dependent transport behavior in the near-threshold regime, the field-effect mobility is extracted under low-drain-bias conditions using transfer characteristics evaluated at constant gate overdrive. This reduces drain-induced electrostatic coupling and helps isolate mobility degradation arising from phonon scattering and trap-related interactions.

#### Field-Effect Mobility vs. Gate Voltage

**Figure 10:**
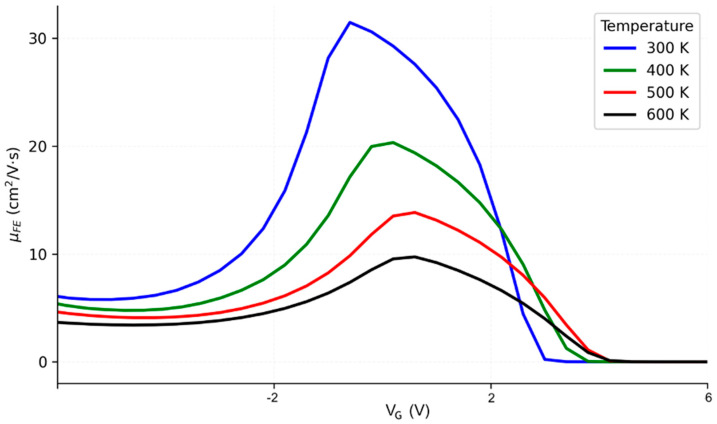
Field-effect mobility μ_FE_ as a function of gate voltage V_G_ for T = 300 to 600 K, extracted from low V_D_ transfer characteristics. The mobility shows a pronounced peak near the threshold region, followed by a reduction at higher gate overdrive. The peak mobility decreases and shifts slightly toward more positive gate voltage with increasing temperature, consistent with enhanced phonon scattering.

The field-effect mobility (μ_FE_) dependence on gate voltage (V_G_) and temperature is presented in Figure 10. In the deep-accumulation regime, μ_FE_ exhibits weak field dependence and follows the expected temperature ordering of 300 K > 400 K > 500 K > 600 K. This trend is consistent with a phonon-limited transport baseline, where increased lattice scattering at higher temperature reduces carrier mobility [41].

As the gate bias approaches the threshold region, μ_FE_ increases and shows a narrow peak slightly below V_th_. This peak arises from the extraction definition (μ_FE_ ∝ g_m_/I_D_) and the rapid onset of channel formation, rather than from a true physical enhancement of carrier mobility. For gate voltages above V_th_, gradual channel pinch-off leads to a reduction in transconductance, and the extracted μ_FE_ decreases accordingly.

The near-threshold peak decreases systematically as temperature increases. This suggests that stronger phonon scattering and temperature-activated interface traps reduce the near-threshold transconductance. To ensure consistent comparison across temperature and interface-trap density, μ_FE_ is additionally evaluated within a fixed gate-voltage window (V_G_ = −4 to −2 V). This selected window was chosen to avoid the sharp near-threshold mobility peak while remaining within the conductive accumulation region of the p-channel device. This means that the extracted value represents the smoother transport region, rather than the sharp peak obtained through (g_m_/I_D_) mobility extraction. In addition, we used a median value to further reduce sensitivity to numerical fluctuations. Finally, we investigated the behavior of neighboring gate-voltage windows for both temperature and D_it_ dependency of mobility, and noticed no change in their trends.

### 3.4. Mobility Degradation Versus Interface-Trap Density and Temperature

**Figure 11:**
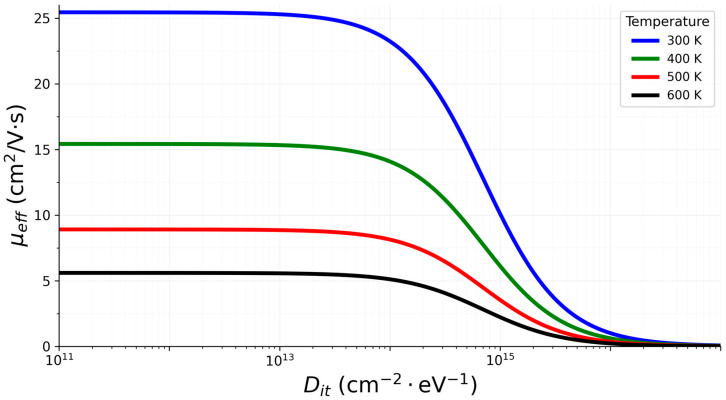
Simulated effective mobility μ_eff_ as a function of interface-trap density D_it_ over a temperature range from 300 to 600 K and V_D_ = −0.1 V. At low D_it_, mobility stays near a phonon-limited plateau. As D_it_ increases to about 10^13^–10^14^ cm^−2^·eV^−1^, the mobility begins to drop sharply. This drop indicates a stronger contribution from interface-trap scattering.

The effective mobility extracted at low V_D_, shown in Figure 11, shows the strong role of interface traps on carrier transport. At each temperature, μ_eff_ stays almost constant at low D_it_, which can be taken as the phonon-limited mobility baseline. Once the trap density increases beyond this regime, μeff begins to roll off, with a clear knee appearing between 10^13^ and 10^14^ cm^−2^·eV^−1^.

At higher trap densities, μ_eff_ approaches a lower mobility limit, suggesting that trap-related scattering strongly limits carrier transport. As temperature increases, the low-D_it_ mobility baseline decreases and the roll-off becomes sharper. This trend is consistent with stronger phonon scattering at elevated temperature, although interfacial charge effects may also contribute. Higher trap occupancy can increase Coulomb scattering near the channel. It can also increase charge-related scattering, making mobility degradation from interface traps stronger at higher temperature.

To describe the mobility degradation with interface-trap density, we used the following semi-empirical expression:

$$\mu_{\mathrm{eff}}\;(T,\;D_{\mathrm{it}})\;\approx \;\frac{\mu_{0}(T)}{1+{\left(D_{it}/D_{0}(T)\right)}^{\beta (T)}},$$

where μ_0_(T), D_0_(T), and β(T) are extracted from the simulation data. The parameter μ_0_(T) represents the low-trap mobility plateau at each temperature. In this regime, transport is mainly controlled by phonon scattering and ionized-impurity scattering, with only a limited contribution from interface traps.

The parameter D_0_(T) gives the density at which the mobility begins to degrade more rapidly. In practice, this is the point where μ_eff_ ≈ 0.5 μ_0_(T). The exponent β(T) controls how sharp the mobility drop is after trap-limited transport takes over.

Before fitting, we applied a monotonic smoothing envelope to the raw μ_eff_ (D_it_, T) data at each temperature. The envelope mainly removes small numerical increases while keeping the low-D_it_ plateau and the post-knee roll-off. The extraction followed three steps. First, we take μ_0_(T) as the median of the initial low-D_it_ points. Next, we assign D_0_(T) where the envelope reaches 0.5 μ_0_(T). Finally, we extract β(T) from the post-knee region using a log-log fit over about the 20–80% decay range.

In Table 1, μ_0_(T), decreases from about 34 cm^2^/V·s at 300 K to about 7.8 cm^2^/V·s at 600 K. That decrease follows the expected drop in the phonon-limited mobility baseline at higher temperature. D_0_(T), however, stays close to 4 × 10^13^ cm^−2^·eV^−1^. For the present oxide/interface system, the onset of trap-limited transport changes only weakly with temperature. The roll-off exponent also stays close to β ≈ 1.3. The main change is in the magnitude of the degradation, not in the curve shape.

The extracted parameters show that the phonon-limited baseline mobility, μ_0_, decreases as temperature increases. In contrast, D_0_ and β show only small change over the same range. The trap-density knee remains nearly stable as the overall mobility level degrades.

D_0_ is the interface-trap density where Coulomb scattering from charged traps becomes comparable to intrinsic phonon scattering. β describes how quickly the device enters the trap-limited regime. Since D_0_ and β vary only slightly with temperature, the temperature dependence is seen mostly as a drop in mobility level.

Interface traps mainly control where the mobility degradation starts and how fast it develops. At higher temperature, phonon scattering controls the mobility baseline. As D_it_ exceeds the transition range, the additional mobility loss is set by the trap density.

The threshold-voltage analysis has shown temperature–trap coupling. Mobility needs separate analysis because phonon scattering and interface-trap-induced scattering both contribute.

For mobility, increasing temperature enhances phonon scattering, which lowers the mobility baseline even when D_it_ is small. When D_it_ is increased, charged interface states introduce additional Coulomb scattering near the surface channel. Because the 2DHG is located within only a few nanometers of the interface, this scattering has a strong effect on transport. At high temperature, changes in trap occupancy can further increase charge-related scattering and modify the local vertical field. The mobility degradation therefore reflects the combined effect of phonon scattering, trap-related Coulomb scattering, and temperature-dependent charge redistribution at the interface.

**Figure 12:**
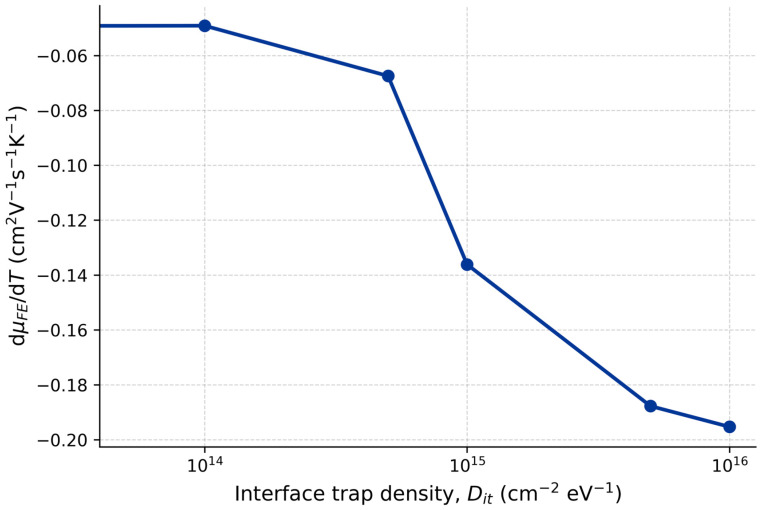
Temperature sensitivity of the field-effect mobility, dμ_FE_/dT, as a function of interface-trap density, D_it_. The decrease in dμ_FE_/dT at higher D_it_ shows that mobility becomes more sensitive to temperature as the interface-trap density increases.

Figure 12 shows how the field-effect mobility changes with temperature at different D_it_ values. The temperature coefficient becomes more negative as D_it_ increases. This means that mobility is more sensitive to temperature when the interface contains more traps. The likely reason is stronger carrier scattering near the channel.

The mobility results extend the V_th_ analysis. Interface traps affect the channel electrostatics, but they also affect carrier transport. In the V_th_ results, the trap effect is mainly related to interface charge and electrostatic control near the channel. For mobility, the same traps also contribute to scattering. However, this effect is weaker because phonon scattering still has a strong influence on transport, especially at higher temperature.

These results show that interface traps affect both V_th_ and mobility. For HD-MOSFETs operating at high-temperature, the two effects should be examined together. Focusing only on one of them may give an incomplete picture of the device behavior.

### 3.5. Gate-Length Dependence of ΔV_th_ Response to Interface Traps

The previous analysis showed that temperature and interface-traps together affect the device. Device geometry can also change this behavior. Specifically, gate length can modify the threshold voltage by changing the channel electrostatics. To study this effect, the gate-length dependence of the interface trap density corresponding to the maximum |ΔV_th_|, D_it_^(ΔVth,max)^, is examined.

Here, D_it_^(ΔVth,max)^ is extracted from the ΔV_th_–D_it_ characteristics for each gate length. This value represents the trap density at which the magnitude of the threshold-voltage shift reaches its maximum under the selected bias and temperature conditions.

**Figure 13:**
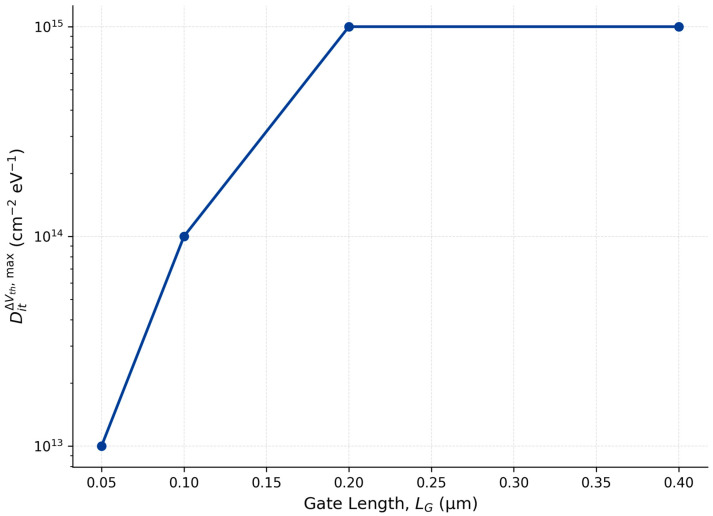
Interface-trap density at maximum threshold-voltage shift, D_it_^(ΔVth,max)^, as a function of gate length L_G_. The characteristic trap density is defined as the D_it_ value at which ΔV_th_ reaches its maximum. In the short-channel range, D_it_^(ΔVth,max)^ increases with L_G_, and then saturates near 10^15^ cm^−2^·eV^−1^ for the longer channels.

Figure 13 shows how D_it_^(ΔVth,max)^ varies with gate length. In the short-channel range, the dependence is clear. The extracted value increases from approximately 10^13^ cm^−2^·eV^−1^ at L_G_ = 0.05 μm to 10^14^ cm^−2^·eV^−1^ at L_G_ = 0.10 μm, and then to about 10^15^ cm^−2^·eV^−1^ at L_G_ = 0.20 μm. For L_G_ = 0.40 μm, the extracted value remains close to 10^15^ cm^−2^·eV^−1^. This apparent saturation suggests that, beyond a certain channel length, the trap density associated with the maximum ∣ΔV_th_∣ becomes less sensitive to gate-length scaling.

The strongest threshold-voltage effect occurs at lower trap densities in shorter-channel devices. For longer channels, a similar effect is reached only after D_it_ is increased. Shorter channels are more sensitive to interface traps, where stronger electrostatic coupling allows interface charge to change the surface potential more directly.

As the gate length increases, D_it_^(ΔVth,max)^ changes only slightly, indicating that the gate-length dependence weakens. For the larger channels, the response is controlled more by D_it_ than by additional geometric scaling.

This dependence on gate-length arises from the electrostatic sensitivity of the surface-transfer-doped channel. For short-channel devices, there is a higher sensitivity to the effect of fringing fields of source-drain and localized interface charges located closer to the gate edges. Thus, even low densities of occupied interface traps can influence the potential profile and result in a threshold-voltage change. That is why the maximum value of |ΔV_th_| is observed for smaller values of D_it_ with decreasing L_G_. However, for longer gate lengths, the portion of the channel beneath the central gate is much less sensitive to the influence of lateral source-drain fields and is dominated by the gate electric field. Thus, a higher density of occupied interface traps is needed to create the perturbation resulting in the maximum threshold-voltage shift. The apparent saturation of the response for longer channels above 10^15^ cm^−2^·eV^−1^ indicates that the limitation of the response is caused by the interfacial charge and its occupation above a certain gate length range.

### 3.6. Implications for High-Temperature HD-MOSFET Design

The simulations lead to three design guidelines for high-temperature HD-MOSFET operation. First, interface-trap control affects both electrostatic stability and carrier transport. At 300 K, keeping D_it_ below approximately 5 × 10^13^ cm^−2^·eV^−1^ helps maintain μ_eff_ close to its phonon-limited baseline and avoids the onset of stronger non-linear shifts in V_th_. The mobility roll-off gives a practical reference point through D_0_. When D_it_ ≈ D_0_, trap-limited transport becomes similar to phonon scattering, and the effective mobility is reduced to roughly half of its low-trap value.

Second, the acceptable interface-trap density range becomes smaller at higher temperature. D_0_ changes only weakly with temperature. The baseline mobility μ_0_(T), however, decreases, leaving less mobility margin as Coulomb scattering becomes dominant. Between 300 and 600 K, the acceptable D_it_ range decreases by roughly one order of magnitude. The mobility curve shape is mainly controlled by the interface-trap distribution. This effect becomes stronger as phonon-limited mobility decreases.

Third, drain-bias conditions also affect the apparent electrostatics. The extracted V_th_ (V_D_) dependence suggests stronger drain–channel coupling at higher temperatures. Operation at reduced ∣V_D_∣, together with appropriate gate length and gate–drain spacing, can help mitigate this drain-induced coupling without compromising the transfer-doped channel.

Beyond threshold-voltage stability, the results also show that interface-trap density can amplify temperature-induced transport degradation. Although carrier mobility in hydrogen-terminated diamond MOSFETs is mainly limited by phonon scattering, high interface-trap densities increase the sensitivity of mobility to temperature. This coupled electrostatic and transport response makes interface engineering an important design factor for high-temperature HD-MOSFET operation, together with diamond material advantages.

To provide perspective on these findings, Table 2 contrasts the current study with previous H-terminated/C-H diamond MOSFET, FET, and 2DHG transport studies as a function of temperature. Prior investigations have primarily shown that their devices could operate at high temperatures, had good temperature stability, or showed temperature-dependent transport properties. Unlike prior work, however, the current study leverages a calibrated TCAD approach to independently change temperature and interface-trap density to investigate their combined effect on (V_th_), (dV_th_/dT), and (μ_eff_).

This comparison highlights the main significance of the present study: rather than only demonstrating high-temperature operation, it quantifies how interface traps modify threshold-voltage stability and mobility degradation over the 300–600 K range.

## 4. Conclusions

We developed a calibrated Sentaurus TCAD model for hydrogen-terminated diamond p-channel MOSFETs and validated it against published transfer characteristics. The model shows good agreement on both linear and semilogarithmic scales, supporting its use for the temperature and trap analyses.

The threshold-voltage shift, ΔV_th_, becomes more negative with increasing temperature and interface-trap density, D_it_, and the dependence is not strictly linear. At low D_it_, the effective mobility μ_eff_ remains near the phonon-limited baseline. It then drops rapidly once D_it_ reaches a transition point, roughly in the 10^13^–10^14^ cm^−2^·eV^−1^ range.

The roll-off exponent β remains nearly unchanged with temperature, suggesting that the interface-trap distribution mainly controls the shape of the mobility degradation. The absolute mobility level, by contrast, is controlled more strongly by phonon scattering. Carrier mobility is affected as well, and the interaction between temperature and traps appears to amplify the temperature-induced degradation.

Keeping the interface-trap density low is crucial for preserving both mobility and threshold stability at room temperature. At elevated temperature, this requirement becomes even more restrictive, since the acceptable trap density is reduced. These findings provide a physics-based framework for evaluating high-temperature operation in hydrogen-terminated diamond MOSFETs. They also offer design guidance for interface engineering and device optimization in ultra-wide-bandgap diamond electronics.

## Figures and Tables

**Table 1:** Parameters extracted from the empirical mobility model between 300 and 600 K. The parameter μ_0_ corresponds to the low-trap mobility plateau, while D_0_ gives the trap density and β describes the roll-off sharpness.

T (K)	μ_0_ (cm^2^/V·s)	D_0_ (cm^−2^·eV^−1^)	β
300	34.23	4.17 × 10^13^	1.31
400	20.98	3.76 × 10^13^	1.31
500	12.02	4.23 × 10^13^	1.30
600	7.78	4.23 × 10^13^	1.30

**Table 2:** Comparison of the present work with representative temperature-dependent H-terminated diamond MOSFET/FET and 2DHG transport studies.

Study	Device/Structure	Temperature Range	Main Reported Result
Kawarada et al. [42]	C–H diamond MOSFET with ALD Al_2_O_3_	RT–400 °C	Demonstrated stable high-temperature operation and high-voltage capability.
Kawarada et al. [43]	C–H diamond MOSFET for power electronics	10–673 K	Reported wide-temperature and high-voltage C–H diamond MOSFET operation.
Peterson et al. [9]	H-terminated diamond 2DHG transport study	25–700 K	Analyzed temperature-dependent mobility and scattering mechanisms.
Oi et al. [44]	Vertical-type 2DHG diamond MOSFET with ALD-induced 2DHG	RT–300 °C	Reported vertical-type 2DHG diamond MOSFET operation and temperature-dependent characteristics up to 300 °C.
Present work	Calibrated TCAD HD-MOSFET with Al_2_O_3_/H-diamond interface	300–600 K	Quantified coupled effects of temperature and D_it_ on V_th_ and μ_eff_.

## Data Availability

The original contributions presented in this study are included in the article. Further inquiries can be directed to the corresponding author.
